# Directional Influence between the Human Amygdala and Orbitofrontal Cortex at the Time of Decision-Making

**DOI:** 10.1371/journal.pone.0109689

**Published:** 2014-10-21

**Authors:** Rick L. Jenison

**Affiliations:** Department of Psychology, University of Wisconsin-Madison, Madison, Wisconsin, United States of America; Centre national de la recherche scientifique, France

## Abstract

There is a growing consensus that the brain makes simple choices, such as choosing between an apple and an orange, by assigning value to the options under consideration, and comparing those values to make a choice. There is also a consensus that value signals computed in orbitofrontal cortex (OFC) and amygdala play a critical role in the choice process. However, the nature of the flow of information between OFC and amygdala at the time of decision is still unknown. In order to study this question, simultaneous local field potentials were recorded from OFC and amygdala in human patients while they performed a simple food choice task. Although the interaction of these circuits has been studied in animals, this study examines the effective connectivity directly in the human brain on a moment-by-moment basis. A spectral conditional Granger causality analysis was performed in order to test if the modulation of activity goes mainly from OFC-to-amygdala, from amygdala-to-OFC, or if it is bi-directional. Influence from amygdala-to-OFC was dominant prior to the revealed choice, with a small but significant OFC influence on the amygdala earlier in the trial. Alpha oscillation amplitudes analyzed with the Hilbert-Huang transform revealed differences in choice valence coincident with temporally specific amygdala influence on the OFC.

## Introduction

There is a growing consensus that the brain makes simple choices by assigning value to the options under consideration, and comparing those values to make a choice. Converging evidence from human fMRI [1]–[14], single unit recordings in non-human primates [15]–[20], and lesion studies [21], [22], suggest that the orbitofrontal cortex (OFC) encodes stimulus value signals at the time of decision that guide choices. Stimulus value signals have also been found in amygdala neurons during simple choices [23], [24], as well as in Pavlovian conditioning paradigms [25]–[27]. However, much less is known about the contribution of the amygdala to the process of making simple choices, or about its interaction with the areas of OFC that compute stimulus values at the time of choice. Neural connections between the amygdala and prefrontal cortex are reciprocal, but the connection density varies considerably [28]–[31]. Connection density based on neural tracers shows that the OFC compared to other areas of the prefrontal cortex receives massive terminations of projections from the amygdala within the superficial cortical layers [29]. Projections from the OFC to the amygdala primarily originate in layer 5, which may pass along information to the amygdala from executive functions in lateral prefrontal cortices that terminate in OFC [32].

Several competing models of the role of amygdala-OFC interactions in simple choice have been proposed. One model states that OFC drives simple choices by computing the values used to identify the best options, and then enhances or inhibits activity in the amygdala associated with compatible or competing Pavlovian responses [33]. In this schema, the stimulus values that drive choices are computed by OFC, and the value related activity in amygdala is tied to competing behavioral controllers. This model predicts that OFC influences amygdala activity at the time of choice, but not the other way around. A second influential view states that amygdala influences the stimulus values computed in OFC [34]–[37]. In this model, amygdala represents the valence and saliency of the stimuli, and modulates value computation activity in OFC, perhaps by increasing attention towards more salient options [38], [39]. This model predicts that amygdala primarily influences OFC activity at the time of choice, but not the other way around. A third view states that amygdala and OFC compute the stimulus values in parallel, with comparable information exchange between amygdala and OFC [24], [40]–[43].

In order to evaluate these competing models, simultaneous local field potentials were recorded from OFC and amygdala in human patients while they performed a simple food choice task. A conditional Granger causality (CGC) analysis was performed to directly test the direction and magnitude of influence. Importantly, evidence for value encoding at the time of decision in OFC has been observed using this specific task, using fMRI [44] and EEG [45], and in amygdala, using single unit recordings [23]. Thus, the task provides an ideal setting to study amygdala-OFC interactions during simple choice. Granger causality [46], [47] has become a prominent technique for inferring the direction of information flow in brain networks from recorded time-series. The basic idea is that if the prediction of a time-series at an instant in time can be improved by the past history of a second time-series, the second time-series is considered to be Granger causal of the first. The time-series can then be swapped to analyze the influence in the opposite direction. Advances in this technology now generalize to conditioning on the influence of all of the ensemble simultaneously recorded time-series, not just pairwise influences, and also generalize into the frequency domain to examine possible synchronous oscillations [48].

## Results

**Figure 1:**
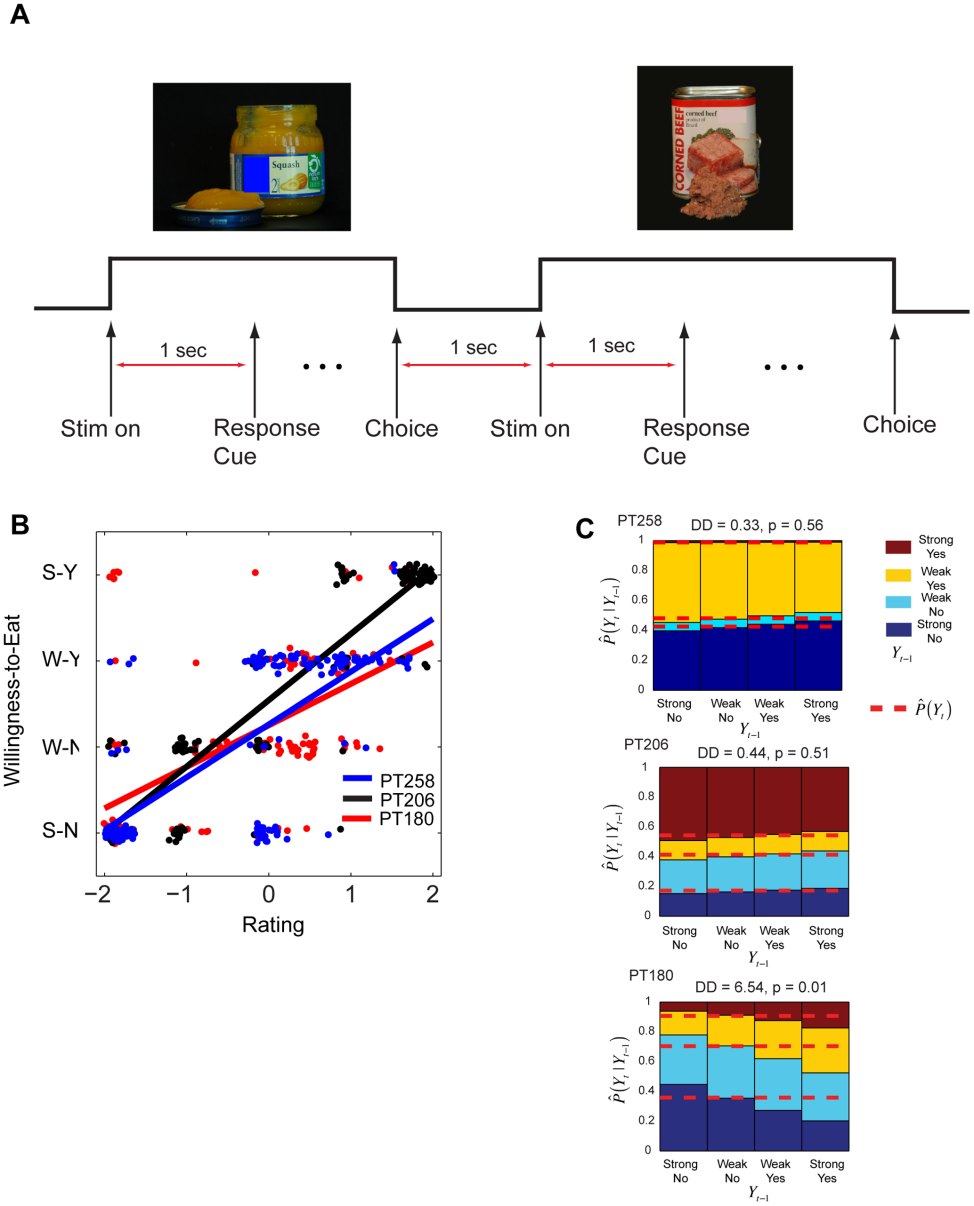
Experimental procedure and behavioral analysis. (A) Task summary. On every trial subjects were shown an image of a snack food for 1 s, at which time they were prompted to indicate whether or not they would be willing to eat the food using a four-item response scale (Strong-Yes, Yes, No, Strong-No). At the end of the experiment one trial was selected at random and the subject's choice was implemented using the actual food. Snacks could be appetitive or aversive, as measured by independent continuous liking-ratings provided by each subject. (B) Scatter plots (jittered) showing the association between prior continuous liking-ratings and choices for each food and subject. Lines correspond to least square fits. Correlation coefficients and p-values were: PT258: 0.73, p<10^−27^; PT206: 0.91, p<10^−63^, PT180: 0.58, p<10^−15^ (C) Estimated cumulative transition probabilities 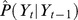 from an ordinal multinomial GLM that conditions choices in trial *t* on the response on the previous trial *t -1*. Dotted lines correspond to the best estimates from a restricted model without the autoregressive (i.e., history independent) component.

The direction of influence between OFC and amygdala at the time of decision-making using a simple food choice task was investigated (Figure 1A). On every trial the subject saw a snack food and had to decide whether they were willing to eat it at the end of the experiment using a four-point scale (Strong-Yes, Weak-Yes, Weak-No, Strong-No). This allowed us to simultaneously measure subjects' choices (yes/no) and the strength of their preference (strong/weak). Foods could be appetitive or aversive, as indicated by the responses in a separate liking-rating task, which provided an independent measure of the value of the different foods for each subject. At the end of the experiment, the choice made in a randomly selected trial was implemented. Local field potential data were recorded during the task simultaneously from amygdala and OFC contacts in three human epileptic patients (labeled PT180, PT206, PT258). See Methods for details.

### Behavioral Analysis

For each subject, a linear regression of choices (1 =  Strong-No to 4 =  Strong-Yes) on the continuous liking ratings (−2 to +2) was estimated in order to verify that subjects computed values with sufficient consistency, and thus did not make choices randomly. As shown in Figure 1B, subjects' choices were highly responsive to the underlying value of the stimuli, with correlation coefficients PT258: 0.73, p<10^−27^; PT206: 0.91, p<10^−63^, PT180: 0.58, p<10^−15^.

A subjects' current choice could be based on choices made in the past, so that the choice made in trial *n* might be influenced by the response on trial *n*-1 [49]–[52]. Therefore the question whether subjects exhibited so-called choice inertia was also investigated. Specifically, an ordinal multinomial generalized linear model (GLM) of the current choice that allowed for an influence of the previous choice (see Methods for details) was fit. As shown in Figure 1C, PT180 exhibited significant choice inertia (p = 0.01), however the other two subjects did not. How this previous trial effect manifests itself in the effective connectivity of the amygdala-OFC network under a simple choice task is unknown, but considered in the electrophysiological results shown below.

### Electrode contact localization

**Figure 2:**
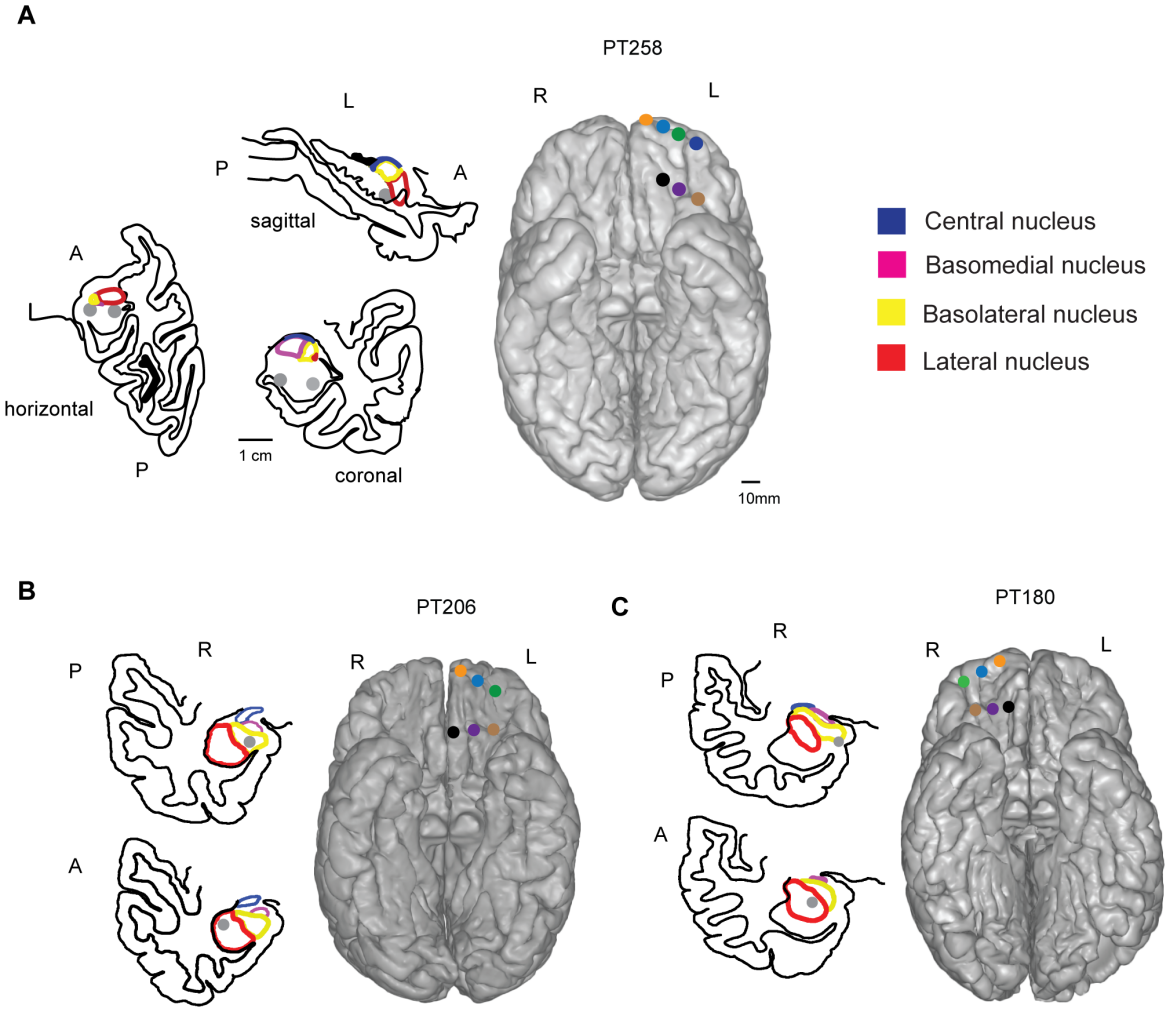
Electrode contact localization. Placements of macrocontacts (gray) relative to amygdala subnuclei and ventral surface of the frontal lobe for each subject (color coded). Coronal plane drawings of the medial temporal lobe (MTL) (anterior [A], posterior [P], left [L] and right [R]) are shown for all three subjects. All three orthogonal planes are shown for PT258.

Figure 2A–C depicts the intracranial location of the contacts for each subject that were localized based on pre- and post-operative high-resolution anatomical scans. All subjects had contacts in or near the lateral nucleus (LA) and basolateral nucleus (BLA). The LA and BLA contacts for PT258 bordered the hippocampus, and consequently all three orthogonal planes are shown for the purpose of localization. For OFC, contacts were associated with cytoarchitectonic areas based on the taxonomies proposed by Ongur and Price [53] and Wallis [54], and spanned Brodmann areas 10, 13, 14 and 47/12.

### Spectral conditional Granger causality analysis

**Figure 3:**
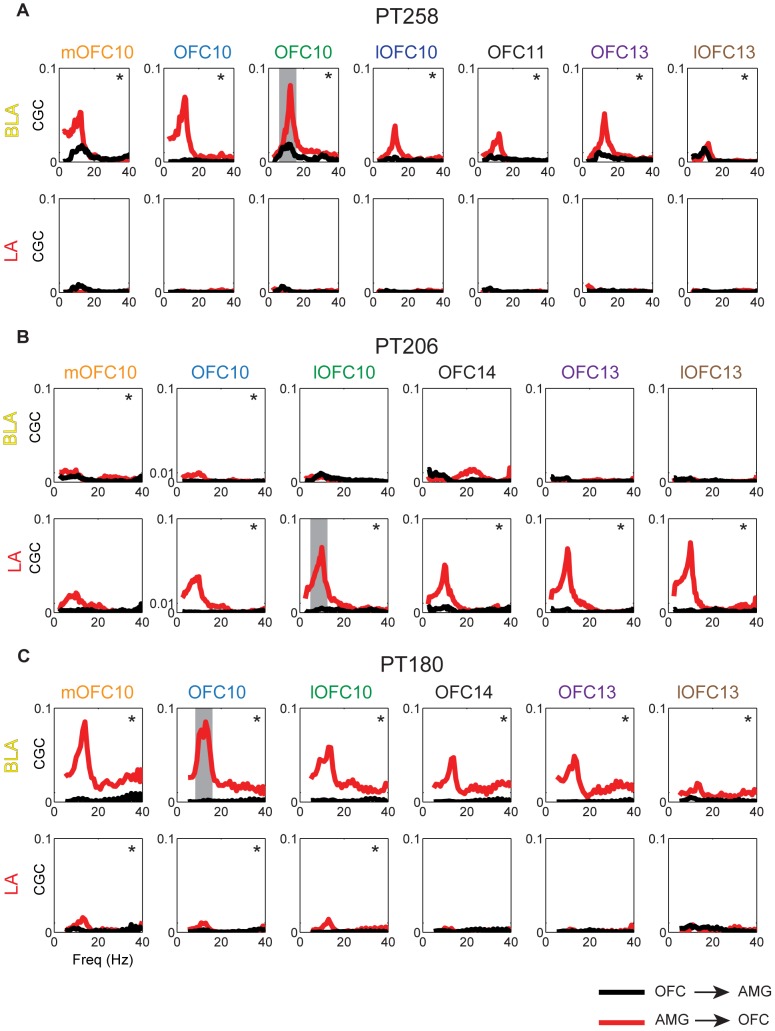
Spectral conditional Granger causality comparisons. (A–C) Spectral CGC between amygdala and OFC contacts, computed over all trials for the interval −1 s to +1 s relative to stimulus onset. The CGC magnitudes as a function of frequency were computed using all contacts shown in Figure 2. Amygdala-to-OFC is shown in red, and OFC-to-amygdala is shown in black. A two-sided cluster-based permutation test for net CGC was performed for each subject by random rearrangement of trials for each contact. For each subject, the null permutation distribution was used to determine the largest-to-smallest net CGC statistic with FWER controlled at.05. Net CGC is given by the CGC from amygdala-to-OFC (red) minus the CGC from OFC-to-amygdala (black). Significant (p<.05) clusters are denoted by *. The maximum cluster (joint contact-pair and frequency) was identified for each subject and is depicted by the gray bar.

Spectral CGC for each amygdala-OFC pair of electrodes over the broad interval −1 to +1 s relative to stimulus onset were computed separately for each contact pair and shown in Figure 3A–C. Spectral CGC for amygdala-to-OFC is shown in red, and OFC-to-amygdala is shown in black. Strong asymmetry on the direction of influence was found: whereas there was sizable influence from either LA or BLA to various regions of OFC, the influence in the other direction was significantly smaller. All contact time-series were explicitly factored into the analysis as a function of the *conditional* nature of the CGC measure [55]. A two-sided cluster-based (contact-pair, frequency dimensions) permutation test was performed for each subject by random rearrangement of trials for each contact (see Methods). Contact pairs yielding a statistically significant net CGC (given by the CGC from amygdala-to-OFC minus the CGC from OFC-to-amygdala) were identified, with the maximum contact-pair frequency band highlighted in gray. The interplay between amygdala and OFC was primarily in the alpha range (8–15 Hz) for all subjects, but it extended to higher frequencies in some cases.

### Time-frequency Coherence analysis

**Figure 4:**
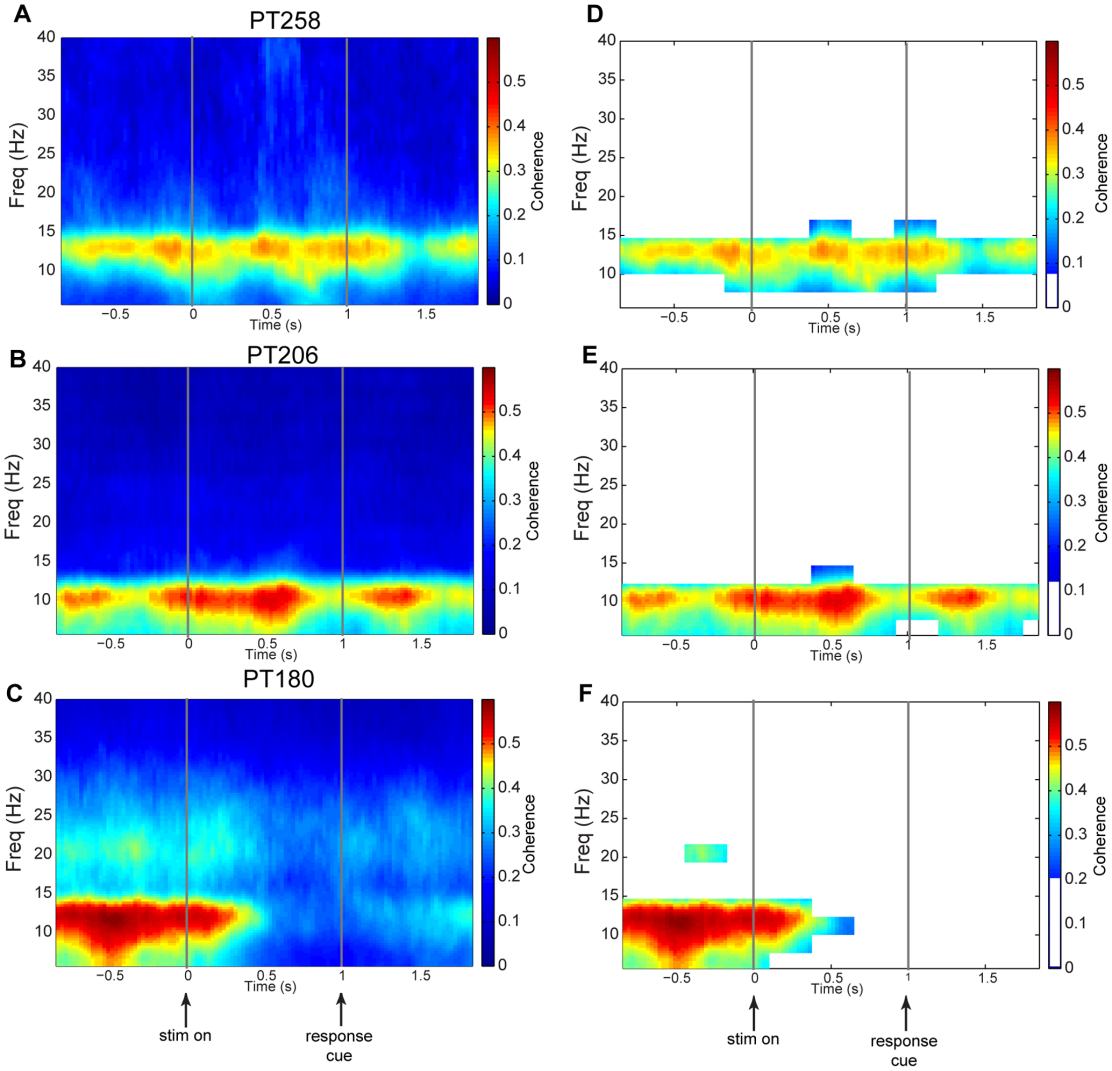
Time-frequency coherence. (A, B, C) Spectral coherence, computed on the interval −1 s to +2 s from stimulus onset and 5 Hz to 40 Hz for each subject. The spectral density used to compute time-frequency coherence was calculated using a sliding 300 ms window and a multitaper technique with a step size of 10 ms. Spectral coherence is only pairwise and is not conditioned on all other contact time series. (D, E, F) A one-sided cluster-based permutation test was performed for each subject by random rearrangement of trials for each contact. The cluster suprathreshold maximum was identified over a (time, frequency) grid composed of 280 ms by 2.5 Hz tiles for a total of 154 frequency-time clusters. The contact-pair direction was analyzed between each of the amygdala-OFC pairs and then aggregated over all of the contact pairs for each subject. The FWER was controlled at.01, and the corresponding critical values were used to mask any clusters that fell within the central region determined by the single null permutation distribution and consequently non-significant.

Next, the local field potential data were analyzed by computing the pairwise spectral coherence (between 0 and 40 Hz) for all pairs of contacts. This provides a necessary initial measure of interaction because, if two areas interact with sufficient strength, we would expect their activity to exhibit some degree of synchronization. Note, however, that the coherence measure is symmetric and non-directional, and thus does not provide information about the direction of information flow. A natural question is whether coherent oscillations fluctuate over the course of a decision trial. To investigate this, coherence on short moving windows over the interval −1 to +2 s relative to stimulus onset was computed together with appropriate significance thresholds and FWER control (see Methods). The pair-wise coherence was analyzed between each of the amygdala-OFC pairs, and then aggregated over all of the amygdala-OFC pairs for each subject. Note that this last step eliminates any bias in choosing a particular contact-pair to analyze, and allows a general assessment of the broader influence between the two areas. As shown in Figure 4 (A, B, C), coherent oscillations between amygdala and OFC in all three subjects were observed, predominantly in the alpha range (8–15 Hz) and localized to specific intervals of time during the task. Figure 4 (D, E, F) show the same time-frequency map with time-frequency clusters masked that failed to reach statistically significant levels, as determined by the cluster-based permutation test.

### Time-frequency Granger causality analysis

**Figure 5:**
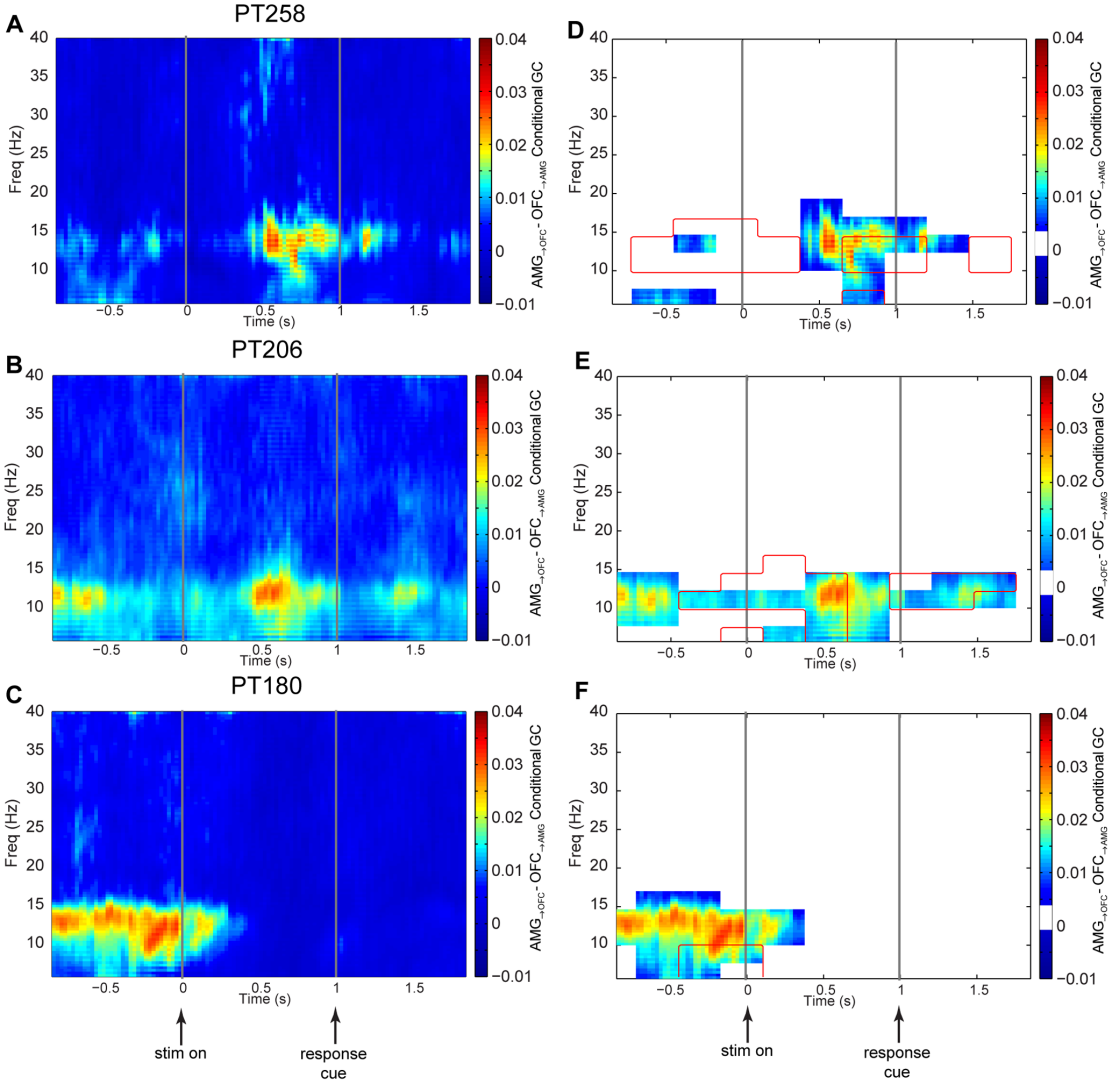
Time-frequency spectral conditional Granger causality. (A, B, C) Net CGC, computed on the interval −1 s to +2 s from stimulus onset and 5 Hz to 40 Hz for each subject. Net CGC is given by the CGC from amygdala-to-OFC minus the CGC from OFC-to-amygdala. The spectral density used to compute CGC was calculated using a sliding 300 ms window and a multitaper technique with a step size of 10 ms. (D, E, F) The contact-pair net direction of influence was analyzed between each of the amygdala-OFC pairs, and then aggregated over all of the amygdala-OFC contacts for each subject. A two-sided cluster-based permutation test was performed for each subject by random rearrangement of trials for each contact. The cluster suprathreshold maximum was identified over a (time, frequency) grid composed of 280 ms by 2.5 Hz tiles for a total of 154 frequency-time clusters. The FWER was controlled using the same method as described for time-frequency coherence, and non-significant clusters masked in an equivalent fashion. A one-sided cluster-based permutation test was also performed on the absolute OFC-to-amygdala CGC to establish its contribution to the net CGC. The red contours define the borders of the clusters, internal to which represents the areas statistically significantly greater than zero, with an internal maximum CGC of 0.010, 0.006, and 0.010 for panels D, E, and F respectively.

In order to investigate the fluctuation of net information flow over the course of a decision trial, net spectral CGC (amygdala-OFC minus OFC-amygdala) was also computed using short moving time windows in a fashion analogous to time-frequency coherence analysis. The contact-pair net direction of influence was analyzed between each of the amygdala-OFC pairs, and then aggregated over all of the amygdala-OFC contacts for each subject, together with appropriate significance thresholds and FWER control (see Methods). Figure 5 (A, B, C) shows the net time-frequency CGC from −1 to +2 s relative to stimulus onset. Figure 5 (D, E, F) again shows the same time-frequency maps but with time-frequency clusters that failed to reach statistical significance masked as determined by the cluster-based permutation test. For all subjects there were significant clusters in the time-frequency grid that revealed a dominant net influence of the amygdala-to-OFC (i.e., with a positive net frequency CGC). However, there was individual variability observed in terms of the timing of influence. PT206 and PT258 had similar patterns of amygdala-to-OFC influence in both timing and frequency range following the onset of the stimulus, prior to the response cue and following the response cue. Furthermore, these patterns were aligned with the coherence results shown in Figure 4, however the timing of amygdala-to-OFC directional influence was noticeably more localized in time compared to coherence measures.

In addition to the computation and two-sided testing of net spectral CGC, the single-sided cluster-permutation was also computed for the OFC-to-amygdala to test whether it was contributing a significant Granger causal effect in this direction. Maintaining FWER control to a.01 level revealed a small, but statistically significant OFC-to-amygdala flow of information localized to specific areas of frequency and time that began prior to the larger flow from amygdala-to-OFC for PT258 and PT206. The significant clusters in time-frequency are identified as red contours in Figure 5 (D, E). This timing suggests that the OFC may activate the amygdala to begin stronger communication toward the OFC, and possibly other regions as well.

Unexpectedly for both coherence and net CGC, evidence for similar direction of information flow was found during the inter-trial period in PT180, and to a lesser degree in PT206 and PT258. As described above, PT180 also exhibited sizable behavioral choice inertia. Neural activity related to previous choices has been observed in a number of brain areas in various species [51], [56]–[58]. A possible explanation for these inter-trial period effects is that, to the extent that the choices must be guided by OFC value signals, the inter-trial modulation from amygdala-to-OFC could provide a mechanism for the behavioral inertia. The amygdala may also be communicating anticipation of a probable value to the OFC that might influence the pending choice. Again, a small but statistically significant time-frequency event was observed for OFC-to-amygdala CGC (Figure 5F), but for this subject it occurred later relative to the start of the large net CGC. This may reflect communication from the OFC to desynchronize activity between these two regions (as shown in Figure 4C).

### Hilbert-Huang Transform Analysis

To identify the presence of any information intrinsic to coherent oscillations between amygdala and OFC at the time of decision-making, LFPs were analyzed using the amplitude of instantaneous frequencies derived from the Hilbert-Huang Transform (HHT) [59]. HHT has recently proven useful in animal and human electrophysiology. For example, Liang and colleagues [60] used this method to show that when a monkey attended to a visual stimulus the amplitude of time-varying gamma oscillations was enhanced compared to when it was not attending to the same stimulus. It was also used in conjunction with Granger causality to examine long-range coupling. The approach was used in the present study to identify any differences between positive (willing to eat) and negative (not willing to eat) valence at the time of decision-making.

**Figure 6:**
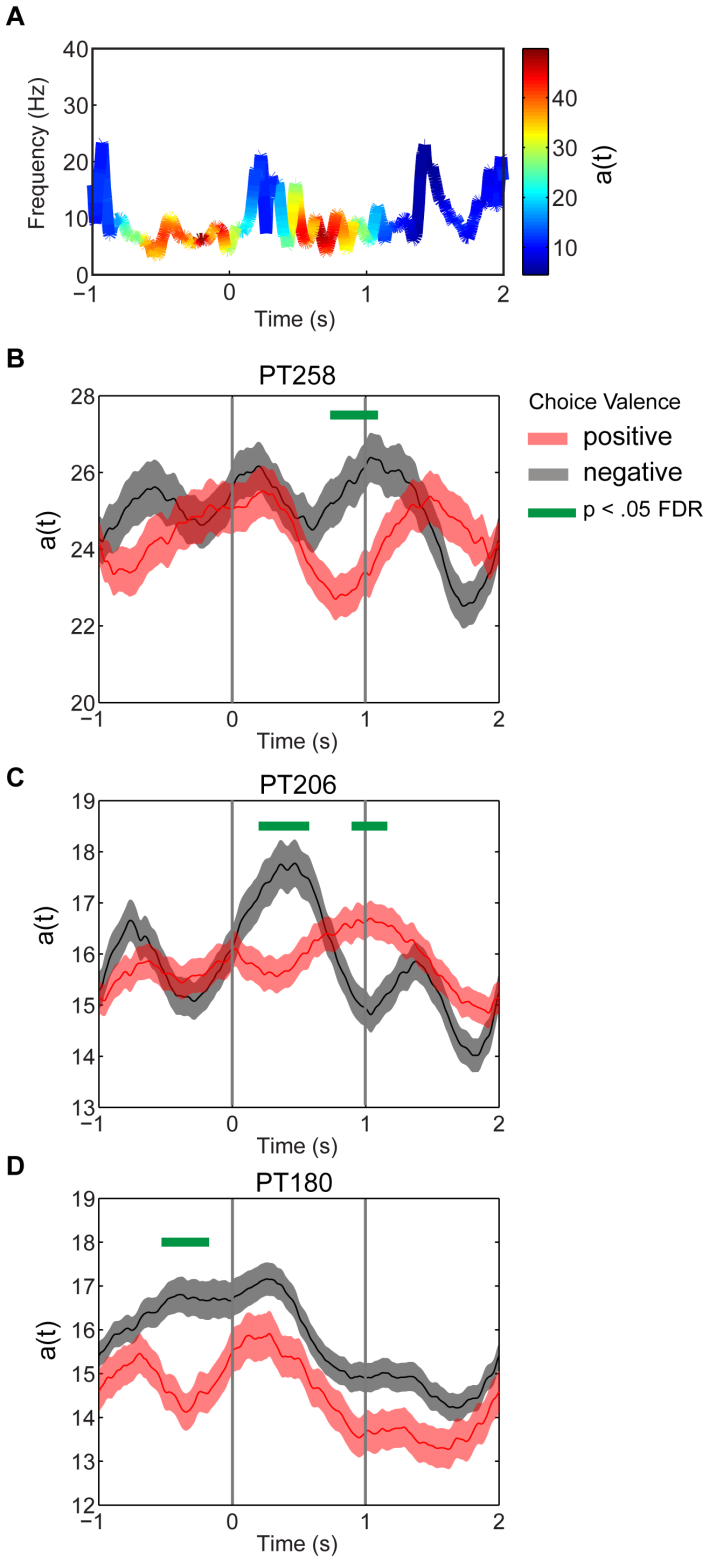
Hilbert-Huang Transform analysis. (A) Illustration of a typical single trial of instantaneous frequency in the alpha range and its amplitude derived from the Hilbert-Huang transform of a recorded LFP on the interval −1 to +2 s from stimulus onset. (B, C, D) Instantaneous amplitude averaged over OFC and amygdala contacts conditioned on valence of choice. Colored lines show mean (+/− s.e.). Green horizontal line denotes significant differences at p<.05 FDR corrected.

The Hilbert-Huang transform was applied to the recorded LFPs from all contacts previously submitted to spectral coherence and CGC analysis. Intrinsic oscillatory mode functions (see Methods) with energy localized to alpha frequency regions were Hilbert transformed to obtain the so-called analytic signal. The analytic signal can be decomposed into instantaneous amplitudes 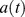 and frequencies 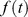 as described in the Methods section. Figure 6 (A) shows a typical Hilbert-Huang transformed single trial with pure, instantaneous dynamic oscillations 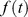 resident in the alpha band. The color represents the instantaneous amplitude 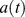 over the course of a single trial. Figure 6 (B, C, D) shows the mean amplitudes aggregated across all amygdala and OFC contacts and collapsed across trials conditioned on the choice valence of each trial. The localized intervals of time where the positive and negative valence means differ significantly (p<.05 FDR corrected) are shown as horizontal green bars. Importantly, the intervals of significant differences occur roughly during those intervals of time when the net CGC is significantly positive and implying amygdala-to-OFC directional influence.

Amplitude differences conditioned on valence were also observed during the inter-trial period leading up to the stimulus onset in PT180. Although mindful that this is a single subject observation, it suggests this directional influence may be more than a non-specific attentional signal, and that these oscillations may indeed bear information for choice in advance of the stimulus onset. One recent human study has shown similar choice bias when no feedback is given and there exists some ambiguity in a sensory stimulus [51]. Model-based fMRI further revealed activation in the frontal eye field associated with increased probability of a repeated choice in the next trial.

## Discussion

A sizable body of previous work has shown that OFC encodes value signals at the time of simple choices (see [61]–[66] for reviews). Previous work by our group has also found value signals in single amygdala units at the time of simple choice [23]. The current study was designed to examine where in time and frequency the OFC and amygdala interact with each other during simple choices. A conditional Granger causality analysis of simultaneous local field potential recordings in OFC and amygdala revealed a stronger direction of influence from amygdala-to-OFC than OFC-to-amygdala. These results suggest that the amygdala modulates the synchrony between itself and the OFC at times coincident with the coding of valence as determined by the Hilbert-Huang transform. Although the direction of information flow was significantly greater for the amygdala-to-OFC, a small but statistically significant flow from the OFC was observed, generally initiated prior to the larger flow of information from the amygdala. This timing could indicate a point of information exchange. One interpretation is that the OFC could be signaling the amygdala to increase its flow of information toward the OFC, as well as to other areas of the brain participating in the computation of value signals. The interplay between amygdala and OFC was primarily in the alpha range (8–15 Hz) for all subjects, but it extended to higher frequencies in some cases. Alpha phase interactions between brain regions have been shown to reflect underlying higher-level attentional, executive, and task-relevant neural processing [67], [68].

A number of competing schemas of the role of amygdala-OFC interactions in simple choice have been proposed. Unfortunately, very little data is available to arbitrate among these models because single unit responses or local field potentials have rarely been recorded simultaneously during a simple choice task independent of training. Furthermore, it is difficult to extrapolate from data collected in other decision-making contexts [33], [69], because an important lesson from these studies is that the nature of the interaction between amygdala and OFC is highly context and task dependent.

The role of amygdala-OFC interactions has received considerable attention in decision contexts other than simple choice. Several lines of previous evidence are consistent with the present study. First, the direction of updating from amygdala-to-OFC is supported by lesion rat studies, which have shown that the amygdala is necessary for learning stimulus-food associations, but not at the time of choice, whereas OFC is necessary at both times [70]. Second, a human fMRI decision-making study showed that patients with amygdala damage exhibit impaired value processing in OFC [71]. Finally, a recent study of amygdala influence on the coding of value in primate OFC showed a decrease in value coding in the OFC upon removal of the amygdala [37]. This primate study is particularly relevant to the current simple choice study in that the association between stimuli and rewards were well established during and following training, and remained constant throughout the study.

However, it is important to emphasize that there is also evidence for the opposite direction of influence in other types of tasks. For example, the present results can be compared with those in [43] that studied OFC-amygdala interactions in a Pavlovian reversal-learning task with both appetitive and aversive unconditioned stimuli. Morrison and colleagues found that after learning stabilized, there were bi-directional influences between amygdala and OFC at the time of the presentation of the conditional stimulus, with a stronger effect on the OFC-to-amygdala direction. This contrasts with the current study, in which the direction of information flow was significantly greater in the amygdala-to-OFC direction than in the reverse. However, early interplay between the OFC and amygdala was observed in the present study, which is consistent with the timing of information flow described in [43].

More generally, electrophysiology studies have shown that both amygdala and OFC encode the value of stimuli during Pavlovian appetitive and aversive conditioning tasks [25]–[27], [38], [72]–[74], and that performance depends on the connectivity between both regions [42]. The amygdala also plays a critical role in updating the value of conditioned stimuli during reversal learning paradigms [75], [76]. In the snake test, where animals have to reach over a plastic snake in order to get a desired food, amygdala and OFC lesions both decrease the force of the associated Pavlovian withdrawal response [77].

A key question for future research is to understand what is the precise computational nature of the amygdala-to-OFC modulatory activity during simple choice. Two alternative (but not necessarily incompatible) models are obvious candidates. First, given that OFC value signals during the course of decision-making are modulated by attention [78], the amygdala might act as a ‘saliency modulator’, inducing OFC to ramp up its processing for stimuli that are particularly relevant [38], [39]. This would improve choices by ensuring that options associated with stronger reward consequences receive more careful processing. Second, amygdala signals might contain specialized information about the value of the stimuli that is passed to OFC to be integrated into the overall stimulus values that guide choices, such as information about their familiarity or history of reward [79], [80]. Evidence from the Hilbert-Huang analysis revealed significant differences between positive and negative valence that coincided with directed influence from the amygdala-to-OFC. It remains to be determined whether these differentially modulated oscillations serve as a value code.

Several limitations of the study are highlighted. First, due to the difficulties, ethics, and limitations of carrying out electrophysiological recordings in human patients, the number of trials is limited. Second, it is clearly not possible to record all relevant processes and pathways that may be driving the amygdala and OFC, which is a fundamental limitation of causality inference from observed data. Thus, the present findings do not speak to other brain areas participating in value computations, or their interactions with the amygdala and OFC. However, given that the available coverage from this study is fully considered in a conditional fashion, the descriptive relations between the measured contacts are provisionally valid [81]. Ongoing studies in our lab that include electrical micro-stimulation of the amygdala and OFC will provide the opportunity to further validate these results by including interventions of the network in the analysis. Granger causality is a well-established statistical method that examines the influence of one or more time-series on another time series, but does not imply cause and effect. Integration of exogenous inputs into these models, such as micro-stimulation or reversible cooling, may bring us closer to a truer causal calculus [82]. Finally, this study focused on a particularly common type of simple choices: approach/avoidance choices over familiar foods. Given that previous studies have shown that OFC encodes stimulus values for a wide class of stimuli (from foods [4] to financial decisions [83], [84] to social exchange [9], [85]), future studies should investigate if the amygdala-OFC interactions identified here also hold for choices involving more complex and/or less familiar stimuli.

## Materials and Methods

### Ethics Statement

The University of Iowa and University of Wisconsin-Madison Institutional Review Boards (IRB) approved the study over the course of data collection and analysis. Informed consent was obtained from each patient after the nature and possible consequences of the studies were explained to them. Patients provided their written informed consent to participate in this study. The original IRB approved signed Informed Consent Document was placed in our research files. A copy of the signed Informed Consent Document was given to the patient, and a copy of the signed Record of Consent form was placed in the patient's electronic medical record. Patients did not incur additional risks by participating in this study. The decision to implant the electrodes, as well as their location, was driven solely by medical considerations.

#### Subjects

Three patients with pharmacologically intractable epilepsy participated in the study (PT180, age 36, female, left-handed; PT206, age 48, male, right-handed; PT258, age 38, male, right-handed;). As part of a surgical treatment for their condition, patients had multi-contact depth electrodes implanted targeting the amygdala, either unilaterally or bilaterally, in addition to subdural low impedance contacts covering the OFC.

#### Task

Subjects participated in two behavioral tasks, always performed in the same order. Both trials involved 81 different snack food items (e.g., chips or candy) widely available at convenience stores throughout the United States. In both tasks the subjects viewed a fixation cross for a variable 750–1250 ms period after which an image of the food item was presented. After a post-image interval of 1000 ms, the subjects were cued to make a response.

In the first task, subjects were shown one of the items on every trial and were asked to provide a liking rating on a continuous sliding scale (anchor: “How much would you like to eat this item at the end of the experiment?”: −2 = dislike very much to +2 =  like very much). Each item was shown once, in random order, for a total of 81 trials. The stimulus set contained both appetitive and aversive items, as demonstrated by the liking ratings. This part of the task provided independent measures of the value of each food, and allowed subjects to become familiar with the experimental set-up and stimuli.

In the second task, subjects were shown one of the items on every trial and had to decide whether or not they would be willing to eat it at the end of the experiment using a discrete four-point scale (Strong-Yes, Weak-Yes, Weak-No, Strong-No). This allowed us to simultaneously measure their choice (yes/no) and their strength of preference (weak/strong). At the end of the experiment one of the 2^nd^ task trials was selected at random and the choice was implemented (i.e., the subject was given the actual item shown in the trial if he responded Strong-Yes/Weak-Yes, and nothing otherwise). The task consisted of two blocks of 81 trials each. Foods were not repeated within a block (see Figure 1A).

### Behavioral test of choice interdependence

The goal of this analysis was to investigate if choice at time *t* (denoted by *Y_t_*) was influenced by the response at time *t-1*, and contributing to the value of the stimuli. For each subject, the following ordinal multinomial generalized linear model (GLM) was fit:
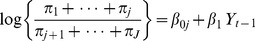
(1)where *J* is the number of response categories (four in this case), β_0j_ is the intercept for the *j* ^th^ response category, and 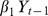 is the autoregressive component corresponding to the subject's choice on the previous trial. The response category probabilities are denoted 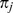, with 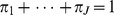. In order to test the null hypothesis of no interdependence, two versions of the model were fitted: one with the autoregressive component and one without it (i.e., with 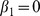). The hypothesis was tested using the difference-of-deviance statistic, which is given by
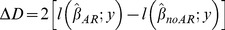
(2)and distributed as 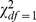. In this formula, *y* is the vector of observed responses, 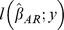 is the maximum likelihood of the estimates of the full model with the autoregressive term, and 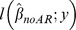 is the maximum likelihood of the estimates of the restricted model.

### Neurophysiological recordings

The recording arrays consisted of 4 platinum-iridium disc electrodes (2.3-mm exposed diameter, 5-mm inter-electrode distance) embedded in a silicon membrane. A subgaleal contact was used as a reference. Simultaneous recordings were obtained from multi-contact hybrid-depth electrodes, stereotactically implanted bilaterally into the medial temporal lobe and amygdala. Recording electrodes remained in place up to 2 weeks under the direction of clinical epileptologists.

### Electrode contact site localization

For each subject a whole brain, high-resolution, T1-weighted structural MRIs (resolution  = 0.78 mm, slice thickness  = 1.0 mm, average of two scans) were acquired, before and after electrode implantation, to determine recording contact locations relative to preoperative brain images. The data were acquired in a Siemens 3T scanner. We also acquired thin-sliced volumetric computed tomography (CT) scans (in-plane resolution  = 0.51 mm, slice thickness  = 1.0 mm) pre- and post implantation. The CT and fMRI data were co-registered using a three-dimensional (3D) linear registration algorithm [86]. Coordinates for each electrode contact obtained from post-implantation CT volumes were transferred to pre-implantation MRI volumes. Results were compared with intraoperative photographs to ensure reconstruction accuracy [86]. OFC contacts were mapped to cytoarchitectonic areas 10 (anterior), 13 (posterior), 14 (medial) and 47/12 (lateral), based on the classification proposed in [53], [54].

#### LFP data preprocessing

Local field potential data were filtered (1.6 to 1,000-Hz band-pass, 12 dB/octave rolloff), amplified, and digitally recorded (original sampling rate 2,034.5 Hz) from low-impedance multicontact subdural grid electrodes (Ad-Tech Medical Instrument, Racine, WI) placed over ventral prefrontal cortex, and from depth electrodes in the amygdala. All recordings were then digitally downsampled to 500 Hz.

### Spectral coherence analysis

The spectral coherence was computed for every pair of electrodes over all trials for a chosen interval. The coherence between simultaneously recorded electrodes *r* and *c* is given by
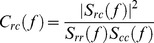
(3)where the cross-spectrum is



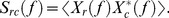
(4)



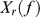
 denotes the Fourier transform of the time-series from the *r^th^* electrode contact and 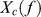 from the *c^th^* electrode contact. The auto-spectrum is obtained when *r* = *c*. Although the cross-spectrum can be estimated directly using the Fourier transform, some form of tapered smoothing is necessary to reduce estimation bias due to the finite length of the dataset. A multitaper technique, in which a set of orthogonal tapers is used to average within data segments, has been proven useful in analyzing non-stationary neural time series [87]. Based on earlier work [88], [89], a system of four orthogonal tapers with prolate spheroidal functions was employed. The spectral and cross-spectral estimates included both averaging over trials as well as tapers, which provides a better (unbiased) method for computing Fourier based spectral Granger causality.

### Spectral conditional Granger causality analysis

In order to investigate the directionality of influence between the amygdala and OFC, a spectral Conditional Granger Causality (CGC) [46], [90]–[96] measure was computed between all pairs of amygdala-OFC electrodes. Intuitively, the CGC tests if activity in a source area can be used to predict subsequent activity on a target area, as should be the case if the source area modulates activity in the recipient area. Importantly, CGC takes into account the predictive effect of all other contacts, which allows us to distinguish between direct influences of interest, and artifactual indirect influences. Note also that CGC need not be symmetric, and thus it allows for identification of directional influences.

A non-parametric spectral approach developed by Dhamala and colleagues [92], [93] was used to compute spectral CGC over a given interval of time. This method has several advantages over more conventional multivariate autoregressive (MVAR) models. First, it permits the direct computation of spectral CGC measures, without having to estimate first the associated MVAR models. This is advantageous because misspecification of the associated MVAR models can lead to spurious findings of CGC, and identifying the correct model order is difficult, particularly under conditions of non-stationarity [97]. Second, the method is multivariate and conditional, in the sense that the simultaneous time series from all electrodes are included in in order to account for direct and indirect influences between contacts. Third, estimates of CGC based on spectral transforms of MVAR models of recorded LFPs are likely to violate key statistical assumptions [94]. In contrast, the Dhamala non-parametric approach sidesteps several of these problems.

As noted above, the cross-spectrum 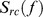 can be estimated directly using the Fourier transform 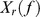 of each time series. Here, trials as well as tapers were averaged, which provided a better (unbiased) method for computing spectral based CGC. The Fourier transform was calculated from 0 to 40 Hz. Inclusion of higher frequencies had no qualitative effect on the results. For the example case of two electrodes, the spectral density matrix is given by



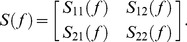
(5)


The diagonal of the matrix contains the auto-spectra and the off-diagonals contain the cross-spectra. This formula can be expanded to include more terms in the case of more than two electrodes.

The spectral density matrix was factored into minimum-phase spectral factors that subserve the intrinsic and causal components of the total power spectrum [98].
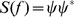
(6)where 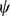 is the minimum-phase spectral density matrix factor and * denotes the conjugate transpose. An expansion of 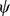




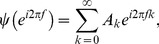
(7)was then used to compute the covariance matrix
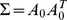
(8)and the transfer function matrix



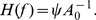
(9)


In the unconditional example of two electrodes, the spectral CGC from the *r^th^* to the *c^th^* electrode is given by



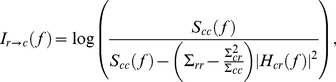
(10)which is the log ratio of total power to intrinsic power [46]. It should be emphasized that in this study the full spectral Conditional GC was computed taking into account the contribution of mediated causality of all electrodes, and is based on a generalization of the expression in Eq. 10.

Routines from the Fieldtrip Toolbox were used for spectral analysis and to factor the spectral matrix [99]. Normal parametric statistical tests for this spectral CGC measure are not available; therefore nonparametric permutation tests are required to compute the necessary null distributions for hypothesis testing.

### Contact-Frequency Cluster Permutation Test

LFP data across trials were randomly rearranged independently for each contact, which destroys the systematic causal relationship between contacts leaving only chance occurrence [94], [96]. The permutation test affords complete freedom in choosing the test statistic, which makes the approach particularly useful for testing differences in directional flow between contacts (net CGC) with no known asymptotic sampling distributions.

The net CGC test statistic was constructed in the following way. First, CGC was computed for all amygdala-to-OFC and OFC-to-amygdala contact pairs, and the net CGC was computed as the simple difference.

The hypothesis that information flows in a dominant directional manner between amygdala and OFC was tested based on spectral CGC computed across contact pairs and frequencies up to 40 Hz. Depending on the available contacts this would result in the neighborhood of a combination of 960 contact-pair by frequency comparisons. Due to the large number of statistical comparisons the family-wise error rate (FWER) inflates, which leads to the multiple comparisons problem. Bonferroni correction is overly conservative and false-discovery-rate correction only weakly controls FWER. To address this problem, a nonparametric strategy originally proposed by Bullmore et al. [100] was followed, and more recently described for testing hypotheses related to spatiotemporal (contact, time) and spectrotemporal (frequency, time) grids by Maris and Oostenveld [101]. The approach is known as cluster-level statistical testing, which uses clusters of neighboring coordinated regions of the analysis grid to reduce the number of independent comparisons. Trials were permuted and the net CGC statistic was calculated, and then the sum of suprathreshold statistics within each cluster were used to generate the mass of the cluster. Finally, the maximum cluster-level statistic over the entire contact-frequency grid was stored as a single null statistic for each permutation step. Because the trials were shuffled, any suprathreshold maximum sum would find itself located in any particular cluster in the grid by chance alone. The p-value of the test statistic is then derived from comparing the resulting null permutation distribution to the single test statistic computed in the same fashion on the original non-permuted data. Note that this approach addresses the multiple comparisons problem by reducing the statistical inference to a single comparison. The cluster-based statistic does to some degree depend on the threshold used to determine which samples on the grid are used to compute the sum in each cluster. The threshold for computing the mass on each cluster of the contact-frequency grid was 2 standard deviations computed from the full set of permuted net CGC values. The threshold choice does not affect the FWER, but it does minimally affect the sensitivity of the test [101].

The spatiospectral (contact-pair, frequency) grid of net CGC values was formed using single contact-pairs by 5 Hz tiles to form either 96 (PT180 and PT206) or 112 clusters (PT258). Net CGC values that exceeded the set threshold were then summed to create the cluster mass [100]. Finally, the single largest cluster mass across the spatiospectral grid was selected as the test statistic to be evaluated against the null permutation distribution. The location of the maximum cluster (contact-pair, frequency) was also recorded.

The permuted distribution was constructed from 1000 steps of random rearrangement and subsequent spectral CGC computation. We were interested in testing information flow in both directions, so a two-sided test was performed using the maximum absolute value as the test statistic. The clustering step was performed separately for positive and negative net CGC permutation samples to test in both tails. The permutation p-value was determined by computing the probability of observing the test statistic value or more extreme based on the null permutation distribution. A p-value for each amygdala-OFC contact-pair was computed for each successive order of clusters (second most extreme, third most extreme, etc.) over all contact-pairs and frequency with FWER controlled at .05 [101], [102].

### Time-frequency spectral coherence analysis

In order to capture changes in coherence within trials, spectral analysis was computed on short moving windows from −1 to 2 s from the stimulus onset. For this application, a 300 ms (containing 150 time points) multitaper moving window was used in time steps of 10 ms. The time series data for all trials were treated as realizations from a common stochastic process. Consequently, the spectral density was averaged over trials and tapers. Prior to time-frequency spectral analysis, the mean at individual time points *across trials* was subtracted from the single trial LFP and then scaled by the standard deviation. Frequencies ranged from 5 to 40 Hz in one Hz steps.

### Time-frequency spectral CGC analysis

In order to capture changes in directed information flow within trials, the non-parametric spectral analysis was repeated on short moving windows from −1 to 2 s from the stimulus onset. The spectral density matrix was factored into minimum-phase spectral factors for each window. Although very short windows are known to lead to biased estimates [95], the combination of multitaper windowing and non-parametric estimation of the spectral density matrix, have been shown to rapidly diminish the problem as the window length is lengthened [92]. Single trial LFPs were preprocessed in a similar manner to that of spectral coherence Time-frequency and spanned the equivalent space specified for spectral coherence.

### Time-Frequency Cluster Permutation Test

A cluster permutation test was performed for each subject by random rearrangement of trials as described above, then time-frequency coherence or net CGC over 1000 permutation steps was calculated. The same cluster-permutation approach as described above was used with the following exceptions: (1) the cluster suprathreshold maximum was identified over a (time, frequency) grid of coherence or net CGC values in 280 ms by 2.5 Hz tiles for a total of 154 time-frequency clusters, and (2) the contact-pair net direction was analyzed between each of the amygdala-OFC pairs and then aggregated over all of the amygdala and OFC contacts for each subject. The FWER was controlled at 0.01, and the corresponding critical values were used to mask any clusters that fell within the central region determined by the single null permutation distribution. The frequency-time clusters that exceeded the 99^th^ percentile were left unmasked. It is important to note that the mask was determined by a single null permutation distribution of maximum (extreme) suprathreshold sums. Cluster-permutation tests are generally more powerful, taking advantage of correlations within clusters, consequently elemental time-frequency samples within the cluster cannot be tested individually [103]. One-tailed time-frequency permutation tests were also performed on the directional spectral CGC on OFC-to-amygdala and amygdala-to-OFC, in addition to the net spectral CGC, in order to test whether the directional magnitudes differ significantly from 0.

Note that unlike spectral CGC, spectral coherence is exclusively pairwise and not conditioned on all other time series. The total dependence between two time-series, which is directly related to spectral coherence, can be decomposed into measures of Granger causality for each direction plus a term that measures the instantaneous interaction, perhaps due to a common driving input [46], [48], [91], [104]. Generally speaking, when the two Granger terms reduce to zero, the total dependence is composed primarily of this instantaneous interaction or correlation. This can explain differences between spectral coherence and Granger causality measures, such as how regions of time-frequency that demonstrate strong coherence can have near zero magnitudes of Granger causality.

### Hilbert-Huang Transform Analysis

This method uses a sifting process to first decompose a time-series into a set of intrinsic oscillatory mode functions (IMFs) having well-defined instantaneous frequencies by empirically identifying the physical time scales intrinsic to the time-series [59]. A signal is considered to be an IMF if the number of its local extrema and the number of its zero crossings is either the same or differ by one. The IMFs were then Hilbert transformed to obtain a meaningful instantaneous frequency and amplitude as a function of time. This method allows for the analysis of non-stationary time-series and provides a better temporal and frequency resolution compared to band-pass filtering followed by a Hilbert transform. Band-pass filtering of the time-series, and then application of the Hilbert transform to extract the instantaneous frequency and amplitude for each passband of interest is not optimal, given that the resulting instantaneous frequencies and amplitudes may not be interpretable, particularly for wider bandwidths [105]. The instantaneous frequency and amplitude are obtained by means of the analytic signal of the j^th^ IMF:
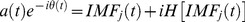
(11)where the Hilbert transform is
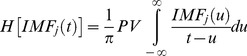
(12)where PV is the principal value of the singular integral [106]. The instantaneous frequency is obtained from the phase as
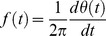
(13)and the instantaneous amplitude is 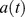, which is the focus of the next section.

A set of IMF signals was step-wise sifted across the frequency range from 0 to 40 Hz and the IMFs spanning the alpha band range were subjected to further analysis. The Hilbert-Huang transform for all samples of 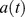 across trials and contacts where collected at each time step t and were conditioned on whether the choice valance was positive (willing to eat) or negative (not willing to eat). The mean and standard deviations were computed at each time point and subjected to a standard two-tailed t-test FDR corrected to identify when in time the two labeled amplitude distributions differ.
